# The novel dual PI3K/mTOR inhibitor NVP-BGT226 displays cytotoxic activity in both normoxic and hypoxic hepatocarcinoma cells

**DOI:** 10.18632/oncotarget.3940

**Published:** 2015-05-14

**Authors:** Carolina Simioni, Alice Cani, Alberto M. Martelli, Giorgio Zauli, Ayman A.M. Alameen, Simona Ultimo, Giovanna Tabellini, James A. McCubrey, Silvano Capitani, Luca M. Neri

**Affiliations:** ^1^ Department of Morphology, Surgery and Experimental Medicine, University of Ferrara, Ferrara, Italy; ^2^ Department of Biomedical and Neuromotor Sciences, University of Bologna, Bologna, Italy; ^3^ Institute for Maternal and Child Health, IRCCS “Burlo Garofolo”, Trieste, Italy; ^4^ Department of Chemical Pathology, Faculty of Medical Laboratory Sciences, University of Khartoum, Khartoum, Sudan; ^5^ Department of Molecular and Translational Medicine, University of Brescia, Brescia, Italy; ^6^ Department of Microbiology & Immunology, Brody School of Medicine, East Carolina University, Greenville, NC, USA; ^7^ LTTA Center, University of Ferrara, Ferrara, Italy

**Keywords:** hepatocellular carcinoma, NVP-BGT226, hypoxia, targeted therapies, PI3K/Akt signaling

## Abstract

Hepatocellular carcinoma (HCC) is one of the most common lethal human malignancies worldwide and its advanced status is frequently resistant to conventional chemotherapeutic agents and radiation. We evaluated the cytotoxic effect of the orally bioavailable dual PI3K/mTOR inhibitor, NVP-BGT226, on a panel of HCC cell lines, since hyperactivated PI3K/Akt/mTOR signaling pathway could represent a biomolecular target for Small Inhibitor Molecules in this neoplasia. We analyzed the drug activity in both normoxia and hypoxia conditions, the latter playing often a relevant role in the induction of chemoresistance and angiogenesis.

In normoxia NVP-BGT226 caused cell cycle arrest in the G_0_/G_1_ phase of the cell cycle, induced apoptosis and autophagy at low concentrations. Interestingly the drug inactivated p-Akt and p-S6 at < 10 nM concentration.

In hypoxia NVP-BGT226 maintained its cytotoxic efficacy at the same concentration as documented by MTT assays and Western blot analysis. Moreover, the drug showed in hypoxia inhibitory properties against angiogenesis by lowering the expression of the transcription factor HIF-1α and of VEGF.

Our results indicate that NVP-BGT226 has a potent cytotoxic effect on HCC cell lines also in hypoxia condition, thus emerging as a potential candidate for cancer treatment in HCC targeted therapy.

## INTRODUCTION

Hepatocellular carcinoma (HCC) is a disease increasing in incidence worldwide [1–5]. One of the most prevalent reasons for the high mortality rate in patients with HCC is the lack of effective treatment, especially for patients with advanced disease [6]. Sorafenib, approved for the treatment of resistant and advanced HCC, has shown low response rate and serious side effects such as hypertension, rash, fatigue, and hand and foot skin reactions [7–9]. For this reason, an effective and well-tolerated pharmaceutical profile for the treatment of advanced HCC is requested to introduce new, potential and therapeutic approaches.

The phosphoinositide 3-kinase (PI3K)/Akt/mTOR signaling pathway is one of the most frequently dysregulated signaling cascades in human malignancies, it displays oncogenic potential and it is implicated in a wide variety of different neoplasms, including HCC [10, 11]. Phosphorylation of Akt activates several substrates, including the mTOR complex 1 (mTORC1), and induces subsequent phosphorylation of downstream targets such as the ribosomal S6 kinase. The activation of mTORC1 results in increased translation and protein synthesis [12]. A second complex comprising mTOR, known as mTORC2, more recently described, appears to act as a feedback loop via Akt phosphorylation on Ser 473 [13].

Given the emerging importance of PI3K/Akt signaling pathway for tumorigenesis in the liver, the potential use of PI3K/Akt pathway modulators is increasingly considered as a targeted therapeutic choice.

Furthermore PI3K/Akt signaling pathway plays also an important role in the regulation of angiogenesis [14, 15], that represents a crucial event in tumor evolution and metastasis. One of the most important stimuli for angiogenesis is hypoxia, that represents a common feature of the tumor microenvironment, since human solid tumors are invariably less well-oxygenated than the normal tissues from which they arose [16]. Sustained hypoxia in a growing tumor is one of the most important stimuli for increased VEGF production and this growth factor, as well as its receptor, is up-regulated in HCC, whose overexpression is inversely related with the prognosis and survival of HCC patients [17].

The frequent activation of the PI3K pathway in cancer and its crucial role in cell growth and survival has made it a much relevant desired target for pharmacologic intervention. The first PI3K pathway-targeted agents approved for cancer treatment were the “rapalogs” Everolimus (RAD001) and Temsirolimus (CCI779), both with the capacity to inhibit the functional protein complex mTORC1. In addition to these agents, other classes of PI3K/mTOR pathway inhibitors have been developed: one is composed by dual inhibitors of PI3K and mTOR (and thus mTORC1 and mTORC2). NVP-BGT226 (BGT226) (Novartis Pharma AG, Basel, Switzerland), an imidazoquinoline derivative, belong to this class being an ATP-competitive dual PI3K/mTORC1/C2 inhibitor: it is a potent pan-class I PI3K inhibitor (p110α, β, δ, and γ, with a preference for the α-isoform -wild type and mutated-) and is a mTORC1/2 catalytic inhibitor [18, 19].

It has been recently demonstrated that PI3K/Akt signaling pathway regulates VEGF and HIF-1α expression, and inhibitors targeting PI3K p110α decrease both VEGF expression and angiogenesis in HCC *in vitro* model [20].

Therefore, in this study, we wanted to investigate the antitumor activity of the orally bioavailable dual PI3K/mTOR inhibitor, NVP-BGT226 (BGT226), on a panel of hepatocellular carcinoma (Mahlavu, SNU475, SNU449, HepG2 and Hep3B) cell lines in either normoxia and hypoxia condition. All these HCC cell lines have an hyperphosporylated Akt, as previously demonstrated by us and by other research groups [21–25]. Mahlavu lack the expression of PTEN and also SNU449 have a low expression of this protein [21, 26].

BGT226 is in phase I/II clinical trials for the treatment of advanced solid tumors, such as breast, head and neck, endothelial cells and lung cancer [11, 18, 26–29] and this is the first work showing the *in vitro* activity of this PI3K/Akt signaling pathway inhibitor in HCC cells. Treatments of HCC cells with BGT226 caused in normoxia condition cell cycle arrest in the G_0_/G_1_ phase of the cell cycle, and induced apoptosis and autophagy at very low doses. Moreover, BGT226 showed in hypoxia conditions inhibitory properties against angiogenesis by inhibiting the expression of HIF-1α and VEGF. Our results indicate that the dual PI3K/mTOR inhibitor, BGT226, is cytotoxic for HCC cell lines in normoxia and in hypoxia condition. It is also a potent inhibitor of the expression of HIF-1α and VEGF and may represent a new promising therapeutic approach in the treatment of hepatocellular carcinoma.

## RESULTS

### BGT226 affects cell viability and is cytotoxic in hepatocarcinoma cell lines

**Figure 1:**
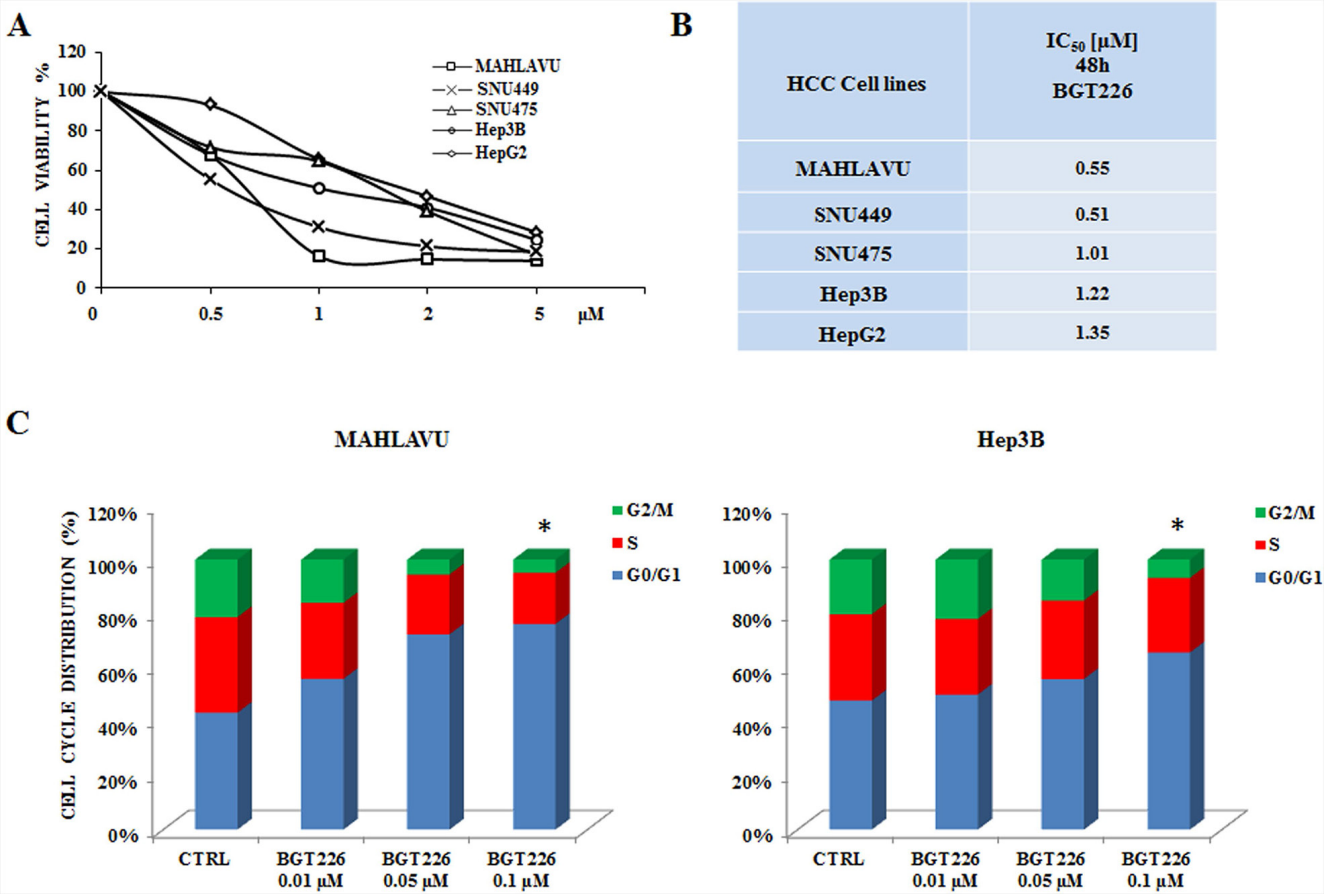
BGT226 affects cell viability and cell cycle in HCC cell lines **A.** MTT assay of HCC cells treated with increasing concentrations of BGT226 for 48 h. SD was less than 8%. **B.** IC_50_ values of BGT226 at 48 h of treatment in Mahlavu, SNU449, SNU475, Hep3B and HepG2 cell lines are reported. **C.** Mahlavu and Hep3B cells were treated with increasing concentrations of BGT226 for 24 h. BGT226 treatment resulted in an increase in cells in the G_0_/G_1_ phase and in a decrease in cells in S and G2/M phase. CTRL, control (untreated) cells. Asterisks indicate significant differences compared with CTRL (*p < 0.05). SD was less than 10%.

To determine whether the dual PI3K/mTOR inhibitor BGT226 could affect the viability of HCC, Mahlavu, SNU475, SNU449, HepG2 and Hep3B cells were incubated in the presence of increasing concentrations of the drug for either 24 or 48 h. Cell viability rates were then analyzed by MTT assays. The experiments documented that already at 24 h all the cell lines were very sensitive to BGT226 (data not shown). After 48 h of treatment cell viability impairment was more evident, with an IC_50_ value ranging from 0.55 μM for Mahlavu to 1.35 μM for HepG2 cells (Figure 1A, 1B). It should be noted that the range of sensitivity is very close and no significant differences are observable among the different cell lines. This observation strengthen the hypothesis that this signaling pathway is altered in a similar way in these cell lines that can be used as a representative panel.

We also investigated the effects of the drug on cell cycle progression. Mahlavu and Hep3B cells were treated for 24 h with increasing concentrations of the drug and stained with Propidium Iodide (PI) for the Muse™ Cell Analyzer. In both cell lines the analysis showed a significant increase in the G_0_/G_1_ phase of the cell cycle (Figure 1C). No significant differences appeared between the activity of BGT226 in Mahlavu and Hep3B cells, being the percentage of cells blocked in G_0_/G_1_ phase very similar.

### BGT226 induces both apoptosis and autophagy

**Figure 2:**
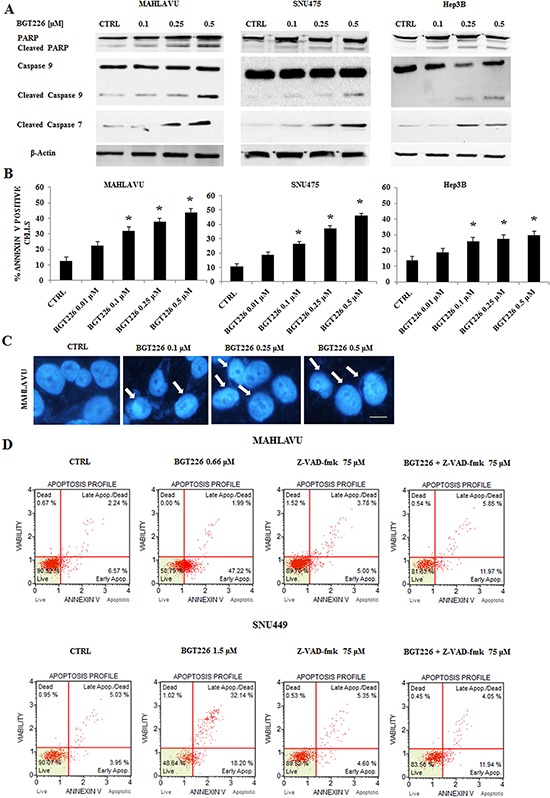
BGT226 induces apoptosis in HCC cell lines **A.** Western blot analysis of Mahlavu, SNU475 and Hep3B cell lines treated for 24 h with increasing concentrations of the drug, ranging from 0.1 to 0.5 μM. Twenty-five μg of protein were blotted to each lane. β-Actin served as a loading control. **B.** Analysis of Annexin-V positive cells after BGT226 treatment using the Muse™ Cell Analyzer in Mahlavu, SNU475 and Hep3B cells. The analysis was performed after 24 h of treatment with increasing concentrations of BGT226. Results are the mean of three different experiments ± SD. Asterisks indicate significant differences compared with CTRL (*p < 0.05). **C.** DNA staining of Mahlavu cells with the fluorescent dye DAPI is reported. In these cells, treated with increasing concentrations of the BGT226 ranging from 0.1 to 0.5 μM, aspects of nuclear chromatin condensation (arrows), representing the apoptotic mode of cell death, are observable. Bar: 10 μm. **D.** Annexin-V analysis after BGT226 treatment, alone and in combination with the pan caspase inhibitor Z-VAD-fmk, in Mahlavu and SNU449 cells. The analysis was performed after 24 h of treatment with BGT226 at the IC_50_value and Z-VAD-fmk at 75 μM.

Previous studies demonstrated that in solid tumors BGT226 can induce apoptosis [11, 30]. In order to establish whether decreased cell viability was related to apoptosis in HCC cell lines, we treated Mahlavu, SNU475 and Hep3B cells for 24 h with increasing concentrations of the drug, and we analyzed the expression levels of PARP, Caspase 9 and the effector Caspase 7. After 24 h of treatment, 0.5 μM BGT226 was able to induce cleavage of PARP, Caspase 9 and Caspase 7 (Figure 2A). We then analyzed apoptosis by Annexin-V staining in Mahlavu, SNU475 and Hep3B cells treated with increasing concentrations of BGT226 for 24 h. The drug induced concentration-dependent apoptosis in all the three cell lines (Figure 2B), with a more relevant effect in Mahlavu and SNU475 than in Hep3B.

After 24 h of treatment with increasing concentrations of BGT226, chromatin condensation was observable in Mahlavu cells by DAPI staining (Figure 2C).

To further analyze whether the intrinsic pathway (*i.e*., caspase-9) was involved in the proapoptotic action of BGT226 in HCC cells, we examined the effect of the pan-caspase inhibitor Z-VAD-fmk, whose activity has already been analyzed in different tumor cell lines [31, 32], administered alone and in combination with BGT226 for 24 h in Mahlavu and SNU449 cells. As shown in Figure 2D, Z-VAD-fmk 75 μM significantly inhibited the cell death induced by BGT226 used at the IC_50_ value, indicating that the apoptotic process mediates BGT226 cytotoxicity.

**Figure 3:**
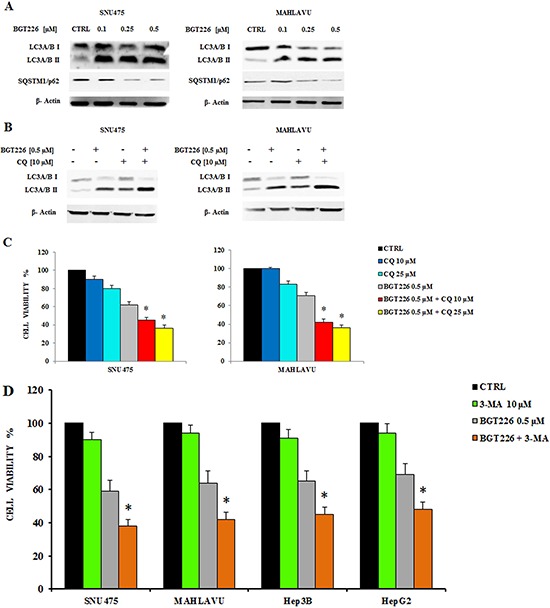
BGT226 induces autophagy **A.** Western blot analysis documenting increased expression of the fast-migrating (lipidated) LC3A/B and decreased expression of SQSTM1/p62 in SNU475 and Mahlavu cell lines treated with BGT226. Twenty-five μg of protein were blotted to each lane. β-Actin documented equal lane loading. **B.** Western blot analysis documenting the effects of chloroquine (CQ) on LC3A/B lipidation in SNU475 and Mahlavu cell lines treated with increasing concentrations of BGT226 for 24 h. β-actin served as a loading control. **C.** MTT assay showing the activity of CQ, alone and in combination with BGT226, in Mahlavu and SNU475 cells after 24 h of treatment. **D.** MTT assay showing the activity of 3-MA, alone and in combination with BGT226, in SNU475, Mahlavu, Hep3B and HepG2 cells after 24 h of treatment. The results are the mean of three different experiments ± SD. Asterisks indicate statistically significant differences with respect to treatment with BGT226 alone (*p < 0.05).

To evaluate whether the treatment with BGT226 could induce autophagy, we analyzed the expression of microtubule associated protein 1 light chain 3 (LC3) A/B I (non lipidated) and its conjugated form LC3A/B II (lipidated) and the expression of another autophagy-related protein, SQSTM1/p62 by Western blot. Lysosomal degradation of autophagosomes leads to a decrease in SQSTM1 levels during autophagy; conversely, autophagy inhibitors stabilize SQSTM1 levels [33]. The levels of LC3A/B II gradually increased whereas p62 decreased in SNU475 and Mahlavu, in a dose dependent manner (Figure 3A), thus showing the activation of autophagy after BGT226 administration.

To verify whether autophagy was either a cell survival or a cell death mechanism, we used the autophagy inhibitor Chloroquine (CQ), since it has recently been reported its ability to block autophagy by inhibiting lysosomal proteases and autophagosome-lysosomal fusion events [34, 35]. SNU475 and Mahlavu cells were treated for 24 h with 0.5 μM BGT226 and 10 μM CQ, alone and in combination, and expression of LC3A/B I-II was assessed by Western blot. BGT226 induced the lipidation of LC3I to LC3II and the addition of CQ further increased LC3A/B II levels (Figure 3B).

We also found that autophagy could protect HCC cells from the cytotoxic effects of BGT226. This was assessed by MTT Assays, after treating SNU475 and Mahlavu cells with 0.5 μM BGT226 and 10 and 25 μM CQ for 24 h. We used BGT226 at the concentration of 0.5 μM to avoid a too high cytotoxicity that would result with a higher concentration of the drug and that would prevent the possibility to observe the additional effect of the two drug combination. CQ, when used alone, displayed only limited cytotoxic effects against SNU475 and Mahlavu cells. However, when it was combined with BGT226, it was possible to detect an increased cytotoxicity in both cell lines (Figure 3C). To further confirm the protective role of autophagy from the cytotoxic effect of BGT226 we also used a different autophagy inhibitor, 3-Methyladenine (3-MA) which blocks at an early stage autophagy by inhibiting the class III phosphoinositide 3-kinase (PI3K) [36]. 3-MA alone did not affect significantly cells after 24 h of treatment. However, when it was combined with 0.5 μM BGT226, it showed an increased cytotoxicity in all HCC cell lines (Figure 3D).

### BGT226 inhibits the PI3K/Akt/mTOR signaling pathway in HCC cells in normoxia and hypoxia conditions

**Figure 4:**
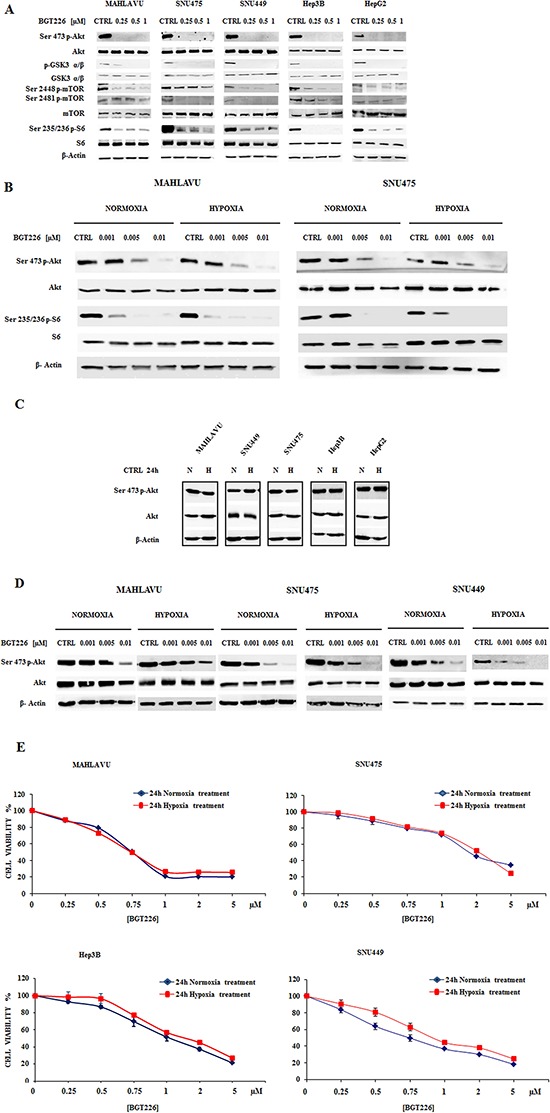
BGT226 modulates PI3K/Akt/mTOR pathway in HCC cells and is sensitive either in normoxia and hypoxia conditions **A.** Western blot analysis for phosphorylated/total Akt, GSK3 α/β, mTOR and its substrate S6 in Mahlavu, SNU475, SNU449, Hep3B and HepG2 cells treated for 1 h with increasing concentrations of BGT226 in normoxic condition. **B.** Western blot analysis for phosphorylated/total Akt and S6 in Mahlavu and SNU475 cells after 1 h of normoxia or hypoxia conditions with the concomitant administration of increasing concentrations of BGT226. **C.** Western blot analysis for phosphorylated/total Akt in HCC cells harvested after 24 h of normoxia or hypoxia conditions. CTRL: control, N: normoxia, H: hypoxia. **D.** Western blot analysis for phosphorylated/total Akt in Mahlavu, SNU475 and SNU449 cells pre-treated for 24 h in normoxia or hypoxia conditions, and then treated for 1 h with increasing concentrations of BGT226. For all the experiments twenty-five μg of protein was blotted to each lane. β-Actin served as a loading control. **E.** MTT Assay showing the activity of BGT226 in HCC cells after 24 h of treatment, in normoxia and hypoxia conditions.

Since in non-small cell lung cancer cell lines the BGT226-dependent modulation of PI3K/Akt/mTOR signal axis has been demonstrated [11], we determined whether this drug also affected downstream signal transduction factors that promote PI3K/Akt/mTOR-mediated cell survival. When the five cell lines were treated with increasing concentrations of BGT226 for 1 h in normoxic conditions, the phosphorylation of Akt and its substrate GSK3 α/β, of mTORC1/2 and of mTORC1 substrate S6 was effectively suppressed in a dose-dependent manner (Figure 4A).

Growth in hypoxia condition can result in a tumor with a more aggressive and malignant phenotype, with a very different sensitivity to drugs of the therapeutic protocol [37–39].

Thus, we first compared the activity of BGT226 in the modulation of PI3K/Akt/mTOR pathway in normoxia or hypoxia conditions with the contemporary administration of the drug at very low concentrations (1-5-10 nM) for 1 h in Mahlavu and SNU475 (Figure 4B). BGT226 was able to suppress both Akt and S6 phosphorylation. It is worth noting the very potent dephosphorylating effect of this drug at < 10 nM concentration and this effect appeared to be independent from the normoxia or hypoxia state of the cells. To better investigate a possible PI3K hyperactivation in hypoxia conditions, we collected the five HCC cell lines after 24 h hypoxia and compared the expression of phosphorylated and total Akt with normoxic cells. As showed in Figure 4C, the expression levels of activated and total Akt in normoxia and hypoxia remained unchanged. To further explore the efficacy of BGT226 in hypoxia, Mahlavu, SNU475 and SNU449 were exposed for 24 h in hypoxia, followed by 1 h treatment with increasing concentrations of BGT226. Results showed that BGT226 was able to suppress Akt phosphorylation also in 24 h hypoxic cells (Figure 4D).

To further assess this observation, MTT assays in four HCC cell lines were performed. Mahlavu, SNU475, Hep3B and SNU449 were incubated in the presence of increasing concentrations of the drug for 24 h, in normoxia or hypoxia conditions. Results showed similar activity of the drug in normoxia as compared with hypoxia (Figure 4E).

### Autophagy is induced by BGT226 in hypoxia condition

**Figure 5:**
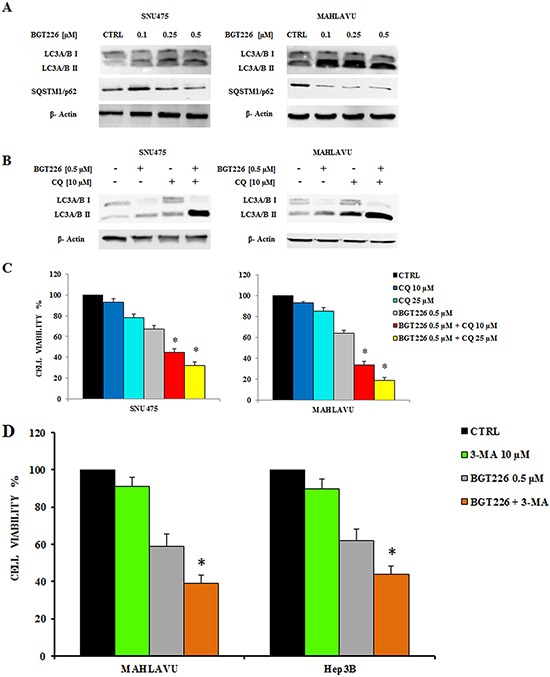
Modulation of autophagy by BGT226 in hypoxia condition **A.** Western blot analysis documenting increased expression of the fast-migrating (lipidated) LC3A/B and decreased expression of SQSTM1/p62 in SNU475 and Mahlavu cell lines treated with BGT226 for 24 h in hypoxia condition. β-Actin documented equal lane loading. **B.** Western blot analysis documenting the effects of chloroquine (CQ) on LC3A/B lipidation in SNU475 and Mahlavu cell lines treated in hypoxia with increasing concentrations of BGT226 for 24 h. β-actin served as a loading control. **C.** MTT Assay showing the activity of CQ, alone and in combination with BGT226, in Mahlavu and SNU475 cells after 24 h treatment in hypoxia condition. **D.** MTT Assay showing the activity of 3-MA, alone and in combination with BGT226, in Mahlavu and Hep3B cells after 24 h treatment in hypoxia condition. The results are the mean of three different experiments ± SD. Asterisks indicate statistically significant differences with respect to BGT226 alone (*p < 0.05).

The administration of BGT226 in hypoxia condition gradually increased the expression levels of LC3A/B II and decreased the expression of SQSTM1/p62 in SNU475 and Mahlavu cells in a dose dependent manner (Figure 5A).

To obtain a comparison with the normoxia data, we also used Chloroquine (CQ) to inhibit autophagy either alone or in combination with BGT226. Thus, SNU475 and Mahlavu cells were treated for 24 h in hypoxia with BGT226 0.5 μM and CQ 10 μM, alone and in combination, and expression of LC3A/B I-II was assessed by Western blot. As shown in Figure 5B, BGT226 induced the lipidation of LC3A/B I to LC3A/B II. The addition of CQ further increased the expression of LC3 A/B II.

Finally, as in normoxia, we also found that autophagy protects HCC cells from the cytotoxic effects of BGT226. This was assessed by MTT assays, after treating SNU475 and Mahlavu cells with BGT226 0.5 μM and CQ 10 and 25 μM for 24 h. CQ alone displayed only limited cytotoxic effects against SNU475 and Mahlavu cells. However, when it was combined with BGT226, it was possible to detect an increased cytotoxicity in both cell lines (Figure 5C). To extend the analysis concerning the protective role of autophagy also in hypoxia we used the autophagy inhibitor 3-MA. The inhibitor alone did not affect cell growth of Mahlavu and Hep3B after 24 h of treatment. However, when it was combined with 0.5 μM BGT226, it was possible to detect an increased cytotoxicity in both cell lines (Figure 5D).

Therefore we were able to assess the same effect of BGT226 also in hypoxia condition and the same role of autophagy in both conditions, normoxia and hypoxia.

### BGT226 inhibits the expression of HIF-1α and VEGF in HCC cells

A low oxygen level is characteristic of solid tumors and represents a negative prognostic factor for cancer patient survival. The response of cancer cells to hypoxia drives neo-angiogenesis but also enhances malignant phenotype development and an increased production of hypoxia-inducible factor (HIF-1), containing HIF-1α and HIF-1β subunits, that acts as a key regulatory transcription factor responsible for adaptive cellular changes. HIF-1α protein is suppressed in cells under normoxic conditions (20–22% O_2_) and its expression is rapidly induced by hypoxic conditions (1–2% O_2_). In humans, HIF-1 has been shown to up-regulate expression of genes affecting a range of target areas of physiology, and it is a major regulator of VEGF, an important growth factor involved in angiogenesis.

**Figure 6:**
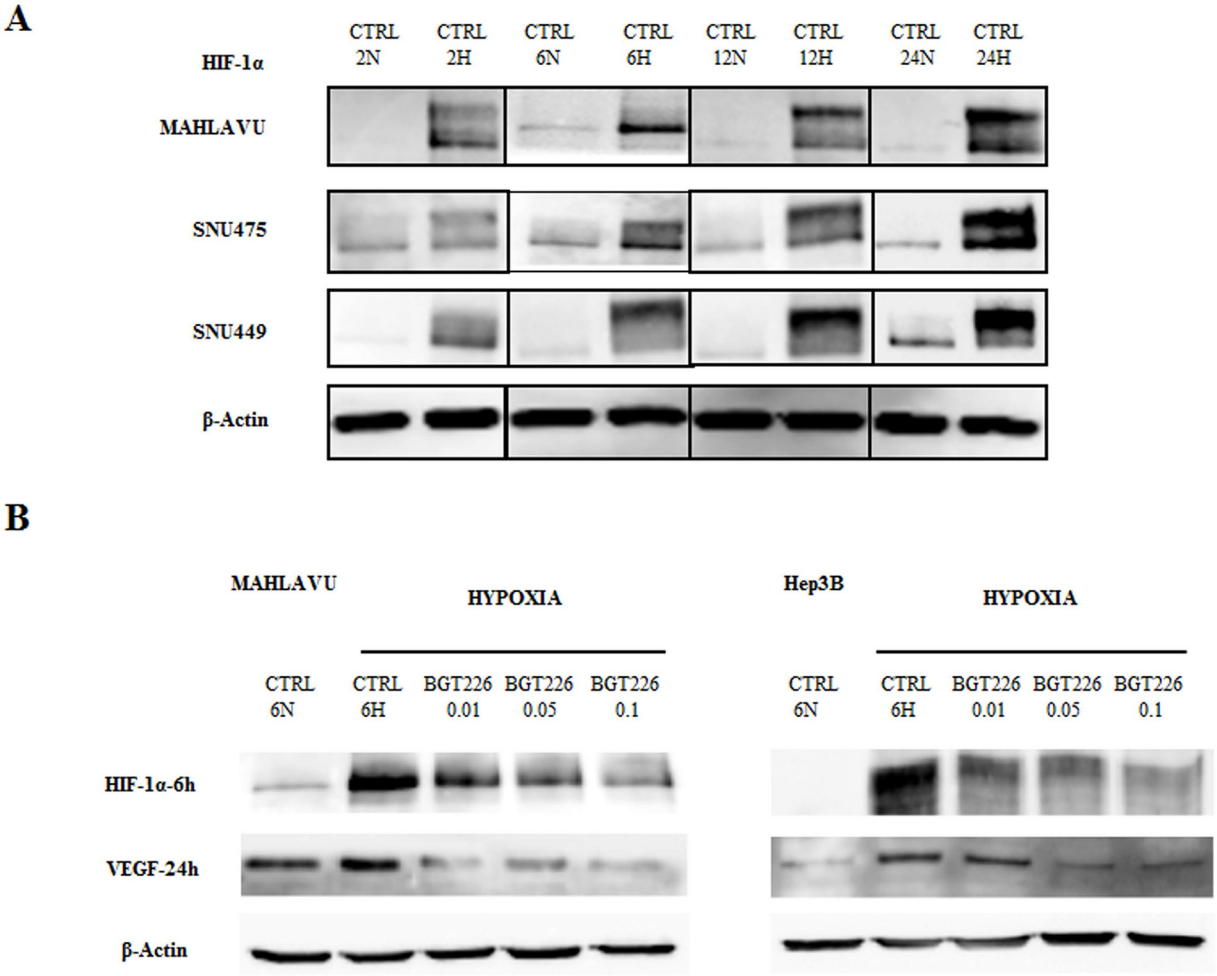
BGT226 and the modulation of HIF-1α and VEGF in HCC cells in hypoxia conditions **A.** Western blot analysis documenting the expression of hypoxia-inducible factor 1α (HIF-1α) in Mahlavu, SNU475 and SNU449 at increasing exposure times of normoxia (N) and hypoxia (H) samples. β-actin served as a loading control. **B.** Western blot analysis showing the expression of HIF-1α and vascular endothelial growth factor (VEGF) in Mahlavu and Hep3B cells treated with increasing concentrations of BGT226. The expression of HIF1α and VEGF is revealed at 6 h and 24 h of hypoxia, respectively. CTRL: control, N: normoxia, H: Hypoxia. β-actin served as a loading control.

Therefore we first evaluated the induction of HIF-1α in HCC cells at increasing times of hypoxia. As shown in Figure 6A, the expression of HIF-1α increased progressively along with exposure to hypoxia.

Treatment with increasing concentrations BGT226 under hypoxic conditions showed a significant, dose dependent inhibition of HIF-1α in Mahlavu and Hep3B cells (Figure 6B). The efficacy of BGT226 in hypoxic condition was further confirmed after treatment of 24 h and detection for VEGF levels. Increasing concentrations of the drug suppressed or reduced the expression of this crucial member of the hypoxia microenvironment (Figure 6B).

## DISCUSSION

The PI3K/AKT/mTOR pathway has a critical role in the pathogenesis of HCC, and it has emerged to be an important therapeutic target for anticancer drug development, since many clinical studies have indicated that activation and deregulation of PI3K/Akt pathway play roles in various human cancers [40, 41].

The panel of HCC cells chosen for this work displays different levels of activated Akt and therefore it is an ideal model for the dual PI3K/mTOR inhibitor NVP-BGT226.

Several mechanisms may be responsible for the activation of PI3K/Akt in HCC. The high frequency of the PI3K p110α gene (PIK3CA) mutations and/or its up-regulation in patients with a shorter survival is responsible for the Akt hyperactivation found in HCC with poor prognosis [42]. Moreover, impaired expression of PTEN could be involved in the regulation of PI3K/Akt activity. Ruan et collaborators demonstrated that overexpression of PTEN significantly reduced the proliferation of HepG2, and the same cell line transfected with PTEN were more sensitive to sorafenib in terms of its ability to inhibit proliferation and to induce apoptosis [43].

A reduction in cell viability was observed in our panel of HCC cell lines treated with low concentrations of BGT226 for 48 h. Among the five tested cell lines, Mahlavu and SNU449 cells were the most sensitive to the drug, with an IC_50_ value of nearly 0.5 μM, even if the difference is not so relevant when compared to Hep3B, being the IC_50_ value of 1.22 μM.

Cell cycle arrest in cancer cells is a major indicator of anticancer activity and has been implicated in different cancers, including HCC [44]. The anti-proliferative effect of BGT226 induced after 24 h cell cycle arrest in the G_0/_G_1_ phase. The cytotoxicity of the drug was mediated by the apoptotic process, as demonstrated by both Annexin V analysis and DAPI-stained samples and with a pan-caspase inhibitor that blocked BGT226-induced apoptosis.

Autophagy is a response to growth limiting conditions, such as nutrient depletion, hypoxia and the presence of cytotoxic drugs [45] and it may trigger increased induction of apoptosis in cells [46]. The correlation between autophagy and tumorigenesis has been explored extensively, but whether autophagy acts as a pro-tumorigenic or anti-tumor player in tumor development and cancer therapy, still has to be fully elucidated [47, 48]. However, autophagy has been found to be associated with drug resistance in HCC [35].

We documented that BGT226 also induced autophagy in HCC cells and its inhibition by CQ and by 3-MA further sensitized HCC cells to the cytotoxic effects of 24 h of BGT226. These findings suggest that in HCC cells autophagy could have a tumor protecting role when neoplastic cells are treated with PI3K/Akt/mTOR inhibitors. Due to these properties CQ has been also studied as a potential agent in cancer therapy, since autophagy could act as a cell-survival pathway in cancer, in agreement with our data [49–51].

In this work we demonstrated for the first time that in HCC models BGT226 has anticancer effects in HCC cells, and these effects act on the proliferation, apoptosis and angiogenesis. Therefore, we hypothesized that its role could not only reflect therapeutic potential in a normoxic condition for tumor cells, but it could also improve therapeutic options for HCC also in a hypoxic microenvironment.

The dual PI3K/mTOR inhibitor, BGT226, has been investigated for the treatment of hematological malignancies, such as acute lymphoblastic leukemias [19, 52], alone, in combination or in comparison with other drugs such as gefitinib or NVP-BEZ235 in different solid tumor models, including non-small cell lung cancer, head and neck squamous cell carcinoma, pancreatic cancer, multiple myeloma and breast cancer [11, 26, 29, 53].

Gefitinib treated cells displayed increased levels of phosphorylation in IGF-1R and Akt, indicating the intensified activation of this cancer-associated signaling pathway in Mahlavu cells [54]. Therefore the efficacy of BGT226 acquires relevance in gefitinib resistant models such as Mahlavu cells.

Since BGT226 has been used with efficacy in this HCC model, it may therefore be considered as a potential therapeutic options in other solid tumors such as colon, ovary or stomach.

Until now, BGT226 activity has been analyzed only in normoxia condition. Therefore in these solid neoplasms BGT226 may have a potential role in developing new therapeutic strategies where the hypoxic conditions could limit drug efficacy.

In this study we observed that BGT226 was capable at very low concentrations to induce Akt and S6 dephosphorylation and also reduced the phosphorylation of mTORC1/2 after 1 h of drug exposure. Interestingly BGT226 maintained these properties also in hypoxia condition.

Tumor hypoxia leads to resistance to radiotherapy and anticancer chemotherapy as well as predisposing for increased tumor metastases [16]. In HCC it has been reported that hypoxia enhances proliferation [55, 56] and suppresses differentiation and apoptosis [57, 58], resulting in tumor malignancy.

Therefore hypoxia cause cellular changes that can result in a more clinically aggressive phenotype and it is of interest the development of therapeutic strategies that can overcome this resistance.

BGT226 appears to be an interesting candidate for this strategy, since it was effective in both normoxia and hypoxia conditions as demonstrated by MTT assay and Western blot analysis with the suppression of cell viability and the inhibition of the PI3K/Akt/mTOR signaling pathway. As expected autophagy in both conditions resulted activated after drug exposure.

HIF-1 plays a central role as the main regulator of the hypoxic transcription response [59, 60]. Increased concentrations of HIF-1 in the proteome of a hypoxic cell result from increased transcription of HIF-1α and HIF-1β genes and decreased HIF-1α protein degradation, an example of hypoxia-mediated post translational control [61, 62]. This transcription factor has been shown to have several transcriptional targets including VEGF, the best-characterized angiogenic growth factor [63].

Our results showed the inhibitory activity of BGT226 in the expression of HIF-1α and VEGF in hypoxia condition. Increasing concentrations of the drug suppressed in a dose dependent manner both these crucial members of hypoxia microenvironment, thus hypothesizing also its potential anti-angiogenic role.

HCC is a highly-vascularized, neoplastic disease with rapid growth and repetitive vascular invasion, and the process of angiogenesis could be a target for novel prognostic and therapeutic approaches to HCC, involving VEGF [64].

In conclusion, BGT226 inhibits the PI3K/Akt/mTOR pathway and shows potent cytotoxic activity by inhibiting cell growth and proliferation in parallel with increasing caspase-dependent and independent apoptosis.

BGT226 may therefore represent a potential anticancer agent in HCC for its capability to target the hyperactivated PI3K/Akt/mTOR signaling pathway and to inhibit, in hypoxia microenvironment, increased translation and synthesis of proteins such as HIF-1α and VEGF, from which may depend tumor progression.

## MATERIALS AND METHODS

### Materials

Dulbecco's modified Eagle's medium (DMEM), RPMI-1640 medium, fetal bovin serum (FBS), nonessential amino acids (NEAA), penicillin and streptomycin were from Lonza (Lonza Milano SRL, Milan, Italy). For cell viability determination, Cell Proliferation Kit I (MTT) was purchased from Roche Applied Science (Basel, Switzerland). Annexin V/7-AAD and cell cycle kits were from Merck-Millipore (Darmstadt, Germany). NVP-BGT226 and Z-VAD-FMK were provided by Selleck Chemicals (Houston, TX, USA). Antibodies to total Akt-1, Ser473 p-Akt-1 and VEGF were from Santa Cruz Biotechnology (Santa Cruz, CA, USA). HIF-1α antibody was provided by BD Biosciences Pharmigen, while all the other antibodies were from Cell Signaling Technology (Danvers, MA, USA), including the rabbit secondary antibody, SQSTM1/p62 (#5114) and Cleaved Caspase-7 (Asp198, #9491) antibodies. The mouse secondary antibody, 3-Methyladenine (3-MA), Chloroquine, 1,4-Diazabicyclo[2.2.2.]octane (DABCO) and 4′,6 diamidino-2-pheny-lindole (DAPI) were from Sigma Aldrich (Milan, Italy). Signals were detected with the ECL Plus reagent purchased from Perkin Elmer (Boston, MA, USA).

### Cell culture and western blot analysis

The HCC cell line Mahlavu was kindly provided by dr. Rengul Cetin-Atalay (Bilkent University, Ankara, Turkey) [65–67] while Hep3B (ATCC no: HB-8064), HepG2 (ATCC no: HB-8065) SNU449 (ATCC no: CRL-2234) and SNU475 (ATCC no: CRL-2236) were obtained from ATCC. Mahlavu, Hep3B and HepG2 were maintained in DMEM medium supplemented with 10% FBS, 2 mM L-Glutamine, 0.1 mM NEAA, 100 U/ml penicillin and 100 μg/ml streptomycin. SNU449 and SNU475 cell lines were maintained in RPMI-1640 medium supplemented with 10% FBS, 2 mM L-Glutamine, 100 U/ml penicillin and 100 μg/ml streptomycin. For normoxic condition, all cells were cultured in a 37°C humidified incubator and an atmosphere of 5% CO_2_ in air. For hypoxic condition, cells were cultured in a CO_2_ incubator (Forma™ Series II Water Jacket CO_2_, Thermo Scientific, USA) maintained at 94% N_2_, 5% CO_2_ and 1% O_2_ for indicated times of treatments. Western blot analysis was performed by standard methods as described elsewhere [68–70].

### Cell viability analysis

MTT (3-[4,5-Dimethylthythiazol-2-yl]-2,5-Diphenyltetrazolium Bromide) assays were performed to assess the sensitivity of cells to drugs, as previously described [71].

### Cell cycle and apoptosis analysis

Cell cycle analysis was performed using the Muse™ Cell Analyzer (Merck Millipore, Milan, Italy). In brief, after 24 h of treatment, cells were harvested, centrifugated at 300 x g for 5 min and washed once with 1X PBS. After fixing them with 70% ethanol for at least 3 h at −20°C, cells were centrifuged at 300 x g for 5 min, washed once with 1X PBS and then 200 μl of Muse™ Cell Cycle reagent was added to each tube with an incubation of 30 min at room temperature in the dark. Samples were then analyzed according to the instrument protocol.

Moreover, analysis of apoptosis was performed by Annexin-V/7-AAD-Assay using Muse™ Cell Analyzer. In brief, cells treated with increasing concentrations of BGT226 were harvested by trypsinization after 24 h of treatment, and a 100 μl cell suspension was labeled for 20 min in the dark with the same volume of the Muse™ Annexin-V & Dead Cell reagent (Merck Millipore). Subsequently, quantitative detection of Annexin-V/7-AAD positive cells was performed with the Muse™ Cell Analyzer.

### DAPI staining

Cell nuclear morphology was evaluated by fluorescence microscopy following DAPI staining. Cells were treated with BGT226 for 24 h in normoxia condition. The cells were washed with PBS (pH 7.4), cytocentrifuged, fixed with 4% paraformaldehyde/PBS and stained for 3 min with 1 μg/ml DAPI. The cells were then washed with PBS, specimens were embedded in glycerol with antifading agent (DABCO) and examined under Zeiss Axiophot fluorescence microscope (Zeiss, Germany).

### Statistical evaluation

The data are presented as mean values from three separate experiments ± SD. Data were statistically analyzed by a Dunnet test after one-way analysis of variance (ANOVA) at a level of significance of *P* < 0.05 vs control samples [72].
